# Charge-Controlled Synthetic Hyaluronan-Based Cell Matrices

**DOI:** 10.3390/molecules23040769

**Published:** 2018-03-27

**Authors:** Patricia S. Hegger, Julia Kupka, Burcu Baykal Minsky, Sabine Laschat, Heike Boehm

**Affiliations:** 1 Department of Cellular Biophysics, Max Planck Institute for Medical Research, 69120 Heidelberg, Germany; patricia.hegger@mpimf-heidelberg.mpg.de (P.S.H.); burcu.minsky@gmail.com (B.B.M.); 2 Department of Biophysical Chemistry, University of Heidelberg, 69120 Heidelberg, Germany; 3 Chemistry Department, University of Stuttgart, 70569 Stuttgart, Germany; Julia.kupka@web.de (J.K.); sabine.laschat@oc.uni-stuttgart.de (S.L.)

**Keywords:** hyaluronan, polyelectrolyte hydrogel, enzymatic degradation, synthetic ECM, cell attachment, glycosaminoglycans, tissue engineering

## Abstract

The extracellular matrix (ECM) represents a highly charged and hydrated network in which different cells in vertebrate tissues are embedded. Hydrogels as minimal ECM mimetics with a controlled chemistry offer the opportunity to vary material properties by varying the negative network charge. In this paper, a synthetic biology model of the ECM based on natural and highly negatively charged polyelectrolyte hyaluronic acid (HA) is characterized with specific emphasis on its charge-related bioactivity. Therefore, the thiol-Michael addition click reaction is used to produce HA hydrogels with defined network structure and charge density. The presented hydrogels show enzymatic degradability and cell attachment. These properties depend on both covalent and electrostatic interactions within the hydrogel network. Furthermore, no unspecific or specific attachment of proteins to the presented hydrogels is observed. In addition, these fundamental insights into charge-related ECM behavior and the influence of electrostatic properties could also lead to innovations in existing biomedical products.

## 1. Introduction

The extracellular matrix (ECM) provides a hydrated, physical scaffold for the different cells in vertebrate tissues. It plays a vital role in different cellular processes such as differentiation, tissue morphogenesis, wound healing, and homeostasis. A synthetic design of ECM model systems is desirable for both biological and medical research purposes as well as for biomedical applications of the resulting biomaterials. However, the task of creating all-encompassing ECM mimetics is fairly complicated due to the diverse, tissue-specific, and dynamic biochemical composition as well as the mechanical properties of native ECMs, which have been altered by age, wounding, and disease as well as through constant reorganization through the embedded cells [1].

**Figure 1:**
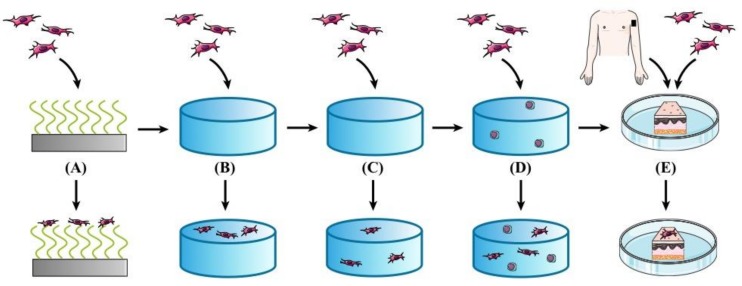
Schematic representation of the progression of extracellular matrix (ECM) mimetics. (**A**) First approaches of coating cell culture plastic with ECM proteins; (**B**) three-dimensional (3D) hydrogels based on synthetic polymers, (**C**) 3D hydrogels made of natural ECM polymers with the possibility of remodeling by cells, (**D**) hydrogels from (C) with incorporated stem cells in order to release their proteins, growth factors, etc., and (**E**) ECM extracted from living donors (Dissertation Patricia Hegger, 2017).

Isolating its three major components: (a) proteins such as collagens, elastins, fibronectins, and laminins [2], (b) polysaccharides such as chondroitin, dermatan, heparin, and hyaluronan (HA), and (c) water, different approaches to fabricating ECM models have been applied in the last three decades. In initial studies, two-dimensional systems consisting of ECM protein-coated cell culture plastics have been used [3]. With the knowledge of the impact of mechanical properties and three dimensions on cell behavior, functionalized polyacrylamide (PA) gels were widely used for remodeling the ECM [4,5]. However, these synthetic networks still neglect the possibility of network reorganization by cells, wherefore hydrogels built up with proteins or other natural ECM components, such as collagen [6] or HA [7,8,9], provided the next stage of development. Hydrogels formed by the biopolymers fibrin, collagen, chitosan, agarose, alginate, or HA [10] are already used in various biomedical applications. They are very well suited to mimic the highly hydrated ECM niche of human and animal tissues due to their high hydrophilicity, low toxicity, and high biocompatibility. However, even these systems display a simplified composition unable to mimic the complex architecture of the natural example. Synthetic ECMs with incorporated proteins, polysaccharides, and growth factors from the pre-cultivation of stem cells in the matrix were developed as more complex and natural systems [11]. Alternatively, taking the risk of immune reactions into account, natural ECMs were also extracted from living donors to serve as skin grafts [12,13] (Figure 1).

Even with advanced laboratory equipment and experimental techniques, mimicking the ECM remains a challenge. Therefore, one of the most promising approaches is to reduce this complex entity to a minimum of components required for specific characteristics or functions of the ECM. With this approach, chemically and physically well-defined as well as reproducible systems can be obtained which can be fine-tuned towards one specific aspect to be studied [14] or one defined application [15,16]. Thus, the models are ideally suited to unravel the impact of different biomolecules or biochemical cues on ECM properties and functions in order to complement and improve materials for biomedical applications.

In this paper, a synthetic biology model of the ECM is characterized with specific regard to its charge-related bioactivity. In this context, the glycosaminoglycan HA, consisting of repeating disaccharides of D-glucuronic acid and *N*-acetyl-d-glucosamine, is used as a highly negative charged component of the natural ECM. It can be crosslinked with short covalent aromatic crosslinkers mimicking desmosine, a natural positively charged ECM component in a previously established hydrogel system [17]. This minimal synthetic ECM model offers the opportunity to regulate the network charge of the material. With this electrostatically well-defined system, the impact of charge on the bioactivity of HA within the ECM can be investigated. Thus, characterizing the effects of the overall hydrogel network charge on enzymatic degradation, protein adsorption, and cell behavior are the first steps in assessing charge regulation in existing, more complex biomaterials for biomedical applications. 

## 2. Results and Discussion

### 2.1. Synthetic ECM Model with Tunable Charge

The natural negatively charged polyelectrolyte hyaluronan (HA), at a defined molecular weight of 74 kDa and different thiolation patterns, was chosen as the main component to fabricate biocompatible hydrogels using the base-mediated thiol-Michael addition click reaction [18]. In addition to differently charged but structurally similar two-armed short pyridinium-based crosslinkers with acrylamide reactive groups, the charge density of these hydrogels can be varied by different degrees of thiolation on the carboxyl groups of the HA [19]. Therefore, the negative network charge of the hydrogels can be calculated as the negative charge of the network structure omitting counter ions [17].

**Figure 2:**
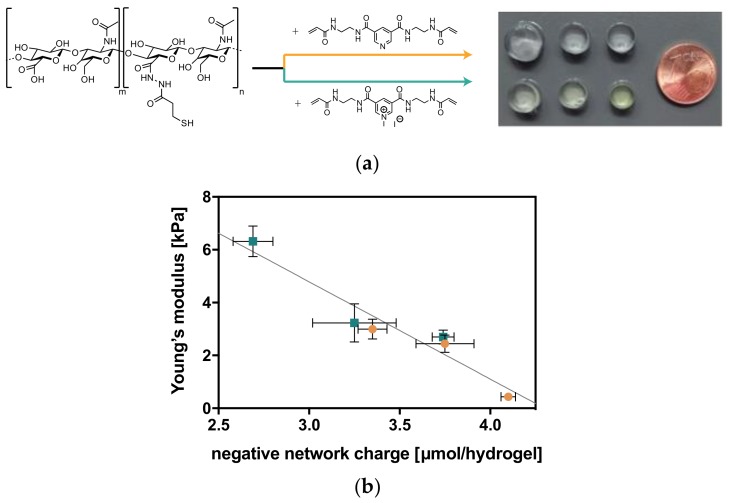
Charge-dependent physicochemical properties of hyaluronan (HA) hydrogels (with degrees of thiolation in % = 18, 25, and 33 and uncharged and charged crosslinker). (**a**) Formed stable HA hydrogels with pyridin-bisacrylamide crosslinker (neutral; top) and pyridinium-bisacrylamide crosslinker (positively charged; bottom) at increasing degrees of thiolation from left to right. Swelling differences are clearly visible in the size of the equilibrium-swollen hydrogels (PBS). (**b**) Linear correlation of stiffness and negative network charge of the presented hydrogel system [17].

Tuning the network charge of the hydrogels leads also to a variation of interconnected physicochemical properties. Generally, the hydrogel properties are regulated by three different types of chemical interactions: (1) covalent linkages within the network, affecting the crosslinker density by variable degrees of thiolation on the HA; (2) electrostatic interactions between crosslinker core and HA backbone mediated by the overall charge of the HA and thus the degree of thiolation as well as the crosslinker charge, and (3) aromatic interactions determined by the different crosslinker core structure. The effects of all three parameters are apparent in different stiffnesses, swelling behavior, and mesh sizes of HA hydrogels with differently thiolated HA and different crosslinkers [17] (Figure 2). All physicochemical parameters are dependent on the negative network charge of the system, which is calculated from the charge contribution of the thiolated HA and the crosslinker per hydrogel, [17] and show a distinct linear correlation. 

Thus, by changing the secondary interactions within the covalently constant network, physicochemical properties can be adjusted. This means that when the number of covalent linkages is kept constant at one degree of thiolation, the physicochemical properties can be adjusted by changing crosslinker charge or crosslinker core. On the other hand, hydrogels with the same network charge can have different stiffnesses, swelling ratios, and mesh sizes by changing the number of covalent links or the crosslinker core.

In the next step, as presented in this paper, the impact of charge density on the biological properties in our ECM mimetics is investigated.

### 2.2. Charge-Dependent Enzymatic Degradation

One important property of HA in the ECM is its biodegradability, allowing for a rapid turnover, especially in wound healing. This process is crucial for enabling cells to move through the highly viscoelastic matrix. Therefore, hyaluronidase IV as a mammalian and hyaluronate lyase as a bacterial enzyme for specific HA degradation are used to assess the stability of the differently charged hydrogels against different types of enzymes. For an easy comparison of the degradation rates, half-lives of different hydrogels in different enzyme solutions are calculated (see Supplementary Materials, Figure S1 and Table S1). Furthermore, control experiments confirmed no unspecific degradation in the solution or by other enzymes (see Supplementary Materials, Figure S2).

#### 2.2.1. Hyaluronidase IV

**Figure 3:**
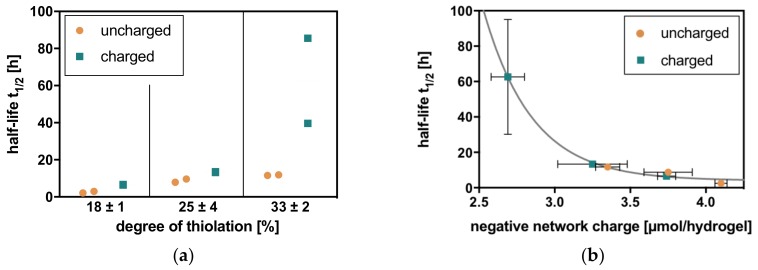
Charge-dependent degradation of HA hydrogels (with degrees of thiolation in % = 18, 25, and 33 and uncharged and charged crosslinkers) with hyaluronidase IV (**a**) follows the general trends of increasing half-life with increasing degree of thiolation and longer half-life with the charged crosslinker compared to the uncharged crosslinker at one degree of thiolation. (**b**) In relation to the negative network charge, the half-lives adopt a first-order exponential decay. Charge dependence can additionally be underlined by the fact that hydrogels with a similar negative network charge show similar half-lives for degradation.

Degradation in hyaluronidase IV solution shows two general trends analogous to the physicochemical properties of these hydrogels [17]. With an increasing degree of thiolation of the HA, the half-lives of the corresponding hydrogels also increase. This correlates with crosslinker density and the rising amount of covalent linkages within the network. At the same degree of thiolation, hydrogels with a charged crosslinker additionally show larger half-lives than comparable hydrogels with an uncharged crosslinker. This relation clearly shows the electrostatic contribution to hydrogel stability, as well as its action against enzymatic degradation (Figure 3).

Half-lives are also clearly correlated to the negative network charge of the hydrogels, resulting in similar half-lives for hydrogels with similar negative network charge. When combined in one graph, a first-order exponential decay of half-life for degradation can be observed with increasing negative network charge (Figure 3).

#### 2.2.2. Hyaluronate Lyase

**Figure 4:**
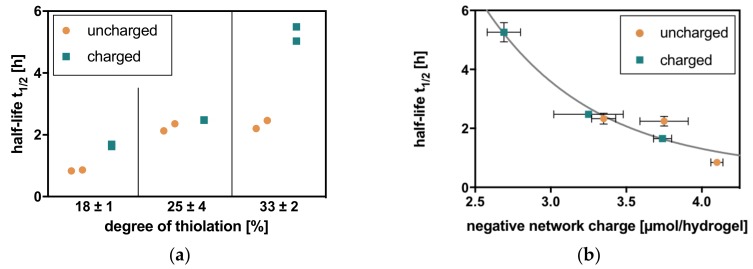
Charge-dependent degradation of HA hydrogels (with degrees of thiolation in % = 18, 25, and 33 and uncharged and charged crosslinkers) with hyaluronate lyase (**a**) follows the general trends of increasing half-life with increasing degree of thiolation and longer half-life with the charged crosslinker compared to the uncharged crosslinker at one degree of thiolation. (**b**) In relation to the negative network charge, the half-lives adopt a first-order exponential decay. Charge dependence can additionally be underlined by the fact that hydrogels with a similar negative network charge show similar half-lives for degradation.

In hyaluronate lyase solution, the behavior of hydrogels is comparable to hyaluronidase IV. Two trends attributed to covalent interactions with an increase in half-life with increasing degree of thiolation and electrostatic interactions with longer half-lives for charged crosslinkers are observed for hyaluronate lyase degradation. Therefore, the correlation for half-lives with negative network charge reveals comparable values for hydrogels with similar negative network charge and a first-order exponential decay for half-lives of hyaluronate lyase degradation with increasing negative network charge (Figure 4).

#### 2.2.3. Comparison

However, the observed degradation in hyaluronate lyase is considerably faster than that in hyaluronidase IV, resulting, for example, in half-lives of 62.6 h in hyaluronidase IV and 5.3 h in hyaluronate lyase in HA hydrogels with a negative network charge of 2.7 µmol/hydrogel. For a better comparison of Michaelis-Menten degradation kinetics, the k-values for both exponential decays of half-lives can be compared. With k = 3.10 for hyaluronidase IV and k = 1.38 for hyaluronate lyase, hydrogels are degraded more than twice as fast in hyaluronate lyase solution.

### 2.3. Charge-Dependent Protein Adsorption

#### 2.3.1. Unspecific

To assess the unspecific electrostatic attachment of ECM proteins to the differently charged HA hydrogels, a bicinchoninic acid assay (BCA assay) is used. In comparison to a phosphate-buffered saline (PBS) blank experiment, no significant adsorption of collagen or fibronectin could be observed in any condition. Thus, the tested proteins cannot adsorb onto hydrogels with differently thiolated HA nor onto hydrogels with differently charged crosslinkers (Table 1 and Supplementary Materials, Figure S3).

#### 2.3.2. Specific

To determine the specific protein adsorption and thus the bioavailability of HA in the different hydrogels, aggrecan as a specific HA binding protein is tested. Similar to collagen and fibronectin, aggrecan could not adsorb onto the presented hydrogels with HA at different degrees of thiolation and differently charged crosslinkers. Thus, seemingly no specific recognition of HA inside the hydrogels is possible for binding proteins, regardless of the number of covalent linkages or the negative network charge of the system (Figure 1 and Supplementary Materials, Figure S3).

### 2.4. Charge-Dependent Cell Attachment

In addition to charge-dependent enzymatic degradation and protein adsorption, the presented interfaces are ideally suited to study electrostatic non-integrin-mediated cell adhesion due to the lack of integrin-specific peptides. Additionally, no cytotoxicity of the presented hydrogels is observed in initial in vitro cytotoxicity tests based on DIN ISO 10993-5 [19]. In this respect, three different cell types are studied to obtain an overview of the charge-dependent cell-hydrogel interactions with additional information on distinct specific HA receptors. The studied cell types are as follows: human dermal lymphatic endothelial cells (HDLEC) with the HA receptor LYVE-1, normal human dermal fibroblasts (NHDF) expressing the HA receptor CD44 and small amounts of RHAMM, and the breast cancer cell line MCF7 presenting CD44 and RHAMM on their cell surface with the ability to express LYVE-1 when metastasizing to the lymphatics [20,21,22]. All three cell types attach to as well as start invading the HA hydrogels and stay alive after 24 h of adhesion time as determined by green/red fluorescent live-dead-staining. The numbers of cells on hydrogels are considerably increased compared to polyethyleneglycole (PEG) passivated glass cover slips (see Supplementary Materials, Figure S5). In comparison to cell culture plastic, however, cells appear more rounded and show smaller focal adhesions, revealing that the hydrogels present a non-optimal interface for cell attachment (see Supplementary Materials, Figure S6).

**Figure 5:**
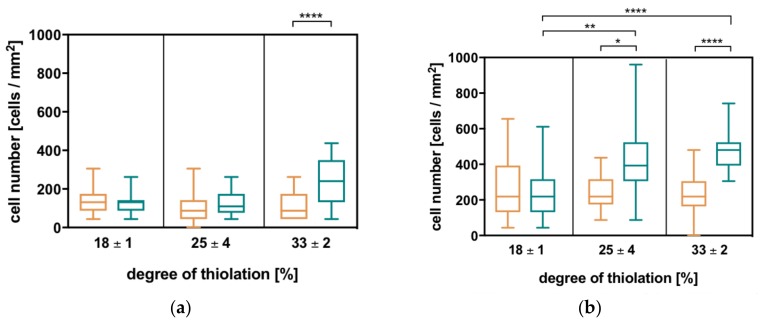

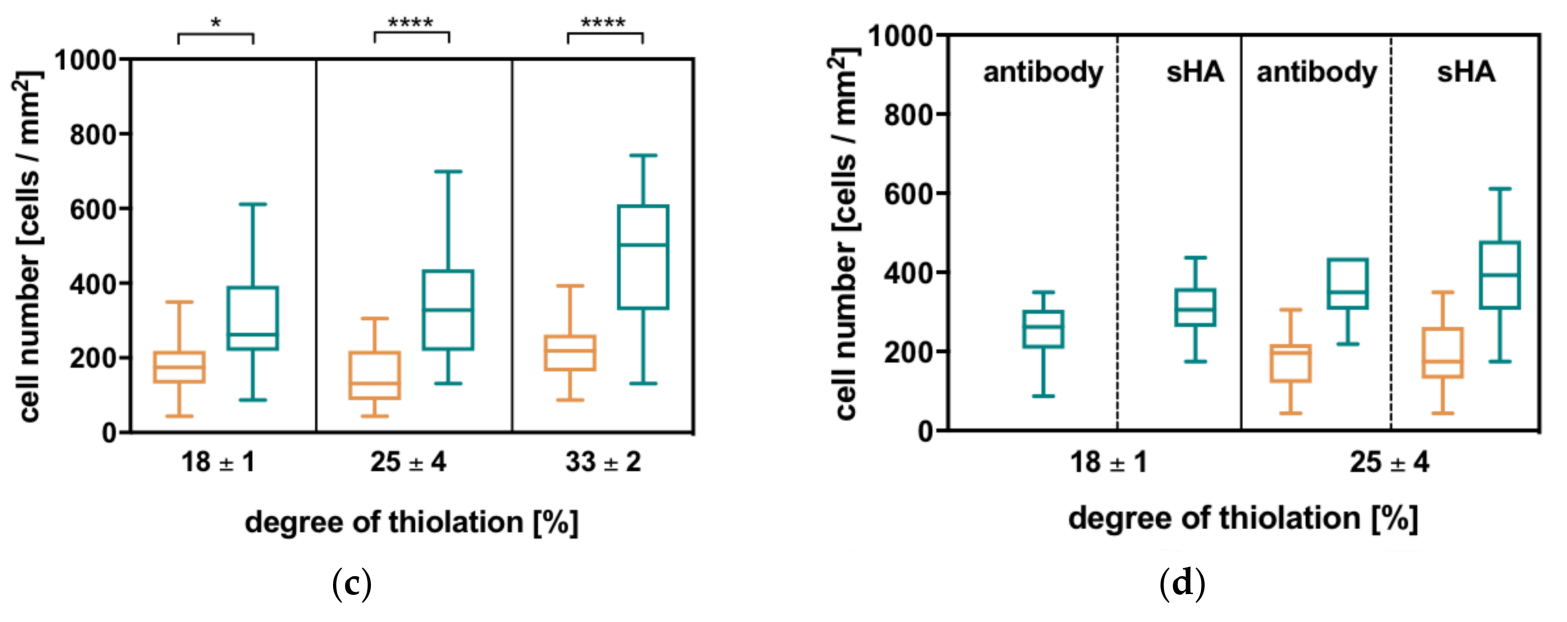
The number of cells adhering to different HA hydrogels with degrees of thiolation in % = 18, 25, and 33 and an uncharged crosslinker (orange) or a charged crosslinker (turquoise) increases with the increasing degree of thiolation and is higher on charged compared to uncharged crosslinker hydrogels. These two general trends are analogous to all other evaluated hydrogel properties and reproducible in all used cell types. (**a**) Human dermal lymphatic endothelial cells (HDLEC), (**b**) the breast cancer cell line MCF7, and (**c**) normal human dermal fibroblasts (NHDF), (**d**) Additionally, blocking CD44 on NHDF with antibody or sHA does not significantly influence the number of attached cells. Statistical analysis is carried out with a Kruskal-Wallis test in combination with Dunn’s multiple comparison for all cell types (*p*-values: **** < 0.0001; ** = 0.001–0.01; * = 0.01–0.1).

By analyzing cell adhesion with respect to different degrees of thiolation and the differently charged crosslinkers, two general trends can be observed for all three cell types: with increasing degree of thiolation and thus the rising number of covalent crosslinks, the number of attached cells increases. At one degree of thiolation, more cells adhere to hydrogels with the charged crosslinker than to the corresponding hydrogels with the uncharged crosslinker, which is much more pronounced in this case compared to the effect of the increasing degree of thiolation (Figure 5). These correspond to the trends observed in the physicochemical properties as well as enzymatic degradation. In comparison to MCF7 and NHDF, HDLEC cells show the previously mentioned trends; however, their numbers on the HA hydrogels remain quite low.

The observed trend shows that the number of attached cells increases with increasing electrostatic interactions within the network. This is further investigated for MCF7 and NHDF since their attachment is more pronounced. By plotting cell number against negative network charge, a linear correlation can be observed. Thus, the number of attached cells decreases with increasing negative network charge (see Supplementary Materials, Figure S7).

In further experiments with NHDF and blocked CD44, the influence of the specific receptor on cell attachment can be ruled out. By blocking CD44 on NHDF cells with the specific receptor antibody, as well as using short HA molecules (molecular weight average = 15 kDa), specific receptor-mediated cell-HA interactions are suppressed. Subsequently, the number of NHDF cells attached to the HA hydrogels with both crosslinkers and different network charges are determined. The result reveals a similar behavior of blocked NHDF on hydrogels compared to untreated cells. The experiment proves that cell attachment is not receptor-mediated and thus purely electrostatic.

## 3. Conclusions

The established ECM mimetics represent a reproducible hydrogel system exhibiting good long-term stability over a period of at least four weeks [17]. Biological characterization reveals two general trends for the different properties corresponding to the previously analyzed physicochemical characteristics which can be tuned by changing the secondary interactions within the network. The ECM mimetics show enzymatic degradability and cell attachment dependent on covalent and electrostatic interactions within the hydrogel network. 

Our experimental results reveal that hydrogels can be specifically degraded by hyaluronan-degrading enzymes whereas they are stable against unspecific degradation. Enzymatic degradability is additionally influenced by the type of enzyme. Hyaluronate lyase degrades the hydrogel faster and the cleavage seems not only influenced by the network charge of the system but also by the nature of the hydrogel. In solution, a reverse trend between hyaluronidase (K_m_ = 0.017 mM) and hyaluronate lyase (K_m_ = 0.12 mM) [23,24] with respect to HA degradation is expected. The interactions of the enzyme active sites with HA seem to be distinct and favor the degradation of immobilized HA within the hydrogel with hyaluronate lyase. This result is especially intriguing considering the equivalent activities of both enzymes indicated by similar degradation rates (in enzyme units) of both hyaluronidase IV and lyase under experimental conditions.

Cell experiments indicated that the overall network charge influences cell attachment prominently. This strongly indicates electrostatically driven, non-receptor-mediated cell-hydrogel interactions. This is especially pronounced when CD44 receptors are blocked on NHDF to exclude a distinct contribution of the specific HA receptors to cell adhesion. Thus, the presented hydrogels offer a platform that supports unspecific but charge-dependent cell attachment.

In conclusion, a hydrogel system as a minimal ECM mimetic with controlled charge is presented to assess the effects of network charge on bioactivity. This system gives the opportunity to vary material properties, especially stability against enzymatic degradation, as well as the attraction of different cells by variation of the negative network charge. Therefore, a two-component (polymer and short crosslinker) system could be established, offering a broad range of different properties to study charge-related bioactivity. In addition to fundamental insights into charge-related ECM characteristics, the influence of electrostatic properties on enzymatic degradability and cell attachment could lead to the innovative incorporation of a defined charge into existing biomedical products.

## 4. Materials and Methods

### 4.1. Materials

Research grade hyaluronic acid (average molecular weight = 15 kDa and 74 kDa) was obtained from Lifecore Biomedical (Chaska, MN, USA). The crosslinkers for hydrogel formation were synthesized according to Reference [17]. The compounds hyaluronidase type IV from bovine testes (LOT: SLBL6343V, 750 U/mg), hyaluronate lyase from *streptococcus pyogenes* (LOT: BCBL3225V, 750 U/mg), fibronectin, aggrecan, and sodium dodecylsulfate were purchased from Sigma-Aldrich (Darmstadt, Germany). Trypsin-EDTA was purchased from Gibco (Waltham, MD, USA) and PLL(20kDa)-g[3.5]-PEG(2kDa) was purchased from SuSoS (Duebendorf, Switzerland). Kits for staining assays and the Pierce™ (BCA) protein assay kit were purchased from Thermo Fisher Scientific (Waltham), and the Calbiochem® Live/Dead Double Staining Kit was procured from Merck (Darmstadt, Germany).

Normal human dermal fibroblasts (NHDF) with fibroblast basal medium (FBM) and supplements (FGM-2 BulletKit) were purchased from Lonza (Basel, Switzerland). Human dermal lymphatic endothelial cells (HDLEC) with endothelial cell growth medium MV2 (EDGM) were purchased from PromoCell (Heidelberg, Germany). The Michigan cancer foundation 7 breast carcinoma cell line (MCF7) and Eagle’s minimum essential medium (EMEM) were purchased from ATCC (Manassas, VA, USA). EMEM supplements fetal bovine serum (FBS), penicillin/streptomycin, non-essential amino acids solution, and sodium pyruvate solution were all purchased from Gibco (Waltham). HCAM CD44 antibody was procured from Santa Cruz (Heidelberg, Germany).

### 4.2. HA Hydrogel Formation and Physicochemical Characterization

#### 4.2.1. HA Thiolation

HA thiolation and subsequent hydrogel formation with the three differently thiolated HA batches (Table 1) was carried out as described in Reference [17,25]. In short, sodium hyaluronate with a molecular weight of 66 to 90 kDas was thiolated with 3,3’-dithiobis(propanoic dihydrazide) at the carboxyl group. With different reaction times, different degrees of thiolation were achieved (Table 2) that were subsequently determined with the colorimetric Ellman’s assay three times in triplicates. For each experiment only one batch of thiolated HA was used in order to prevent batch-to-batch variations [17].

#### 4.2.2. HA Hydrogel Formation and Determination of Reacted Thiols

All used solutions were degassed for 15 min in an ultrasonic bath to avoid disulfide bond formation. The thiolated HA (HA-SH) was dissolved in borate buffer (pH = 10.0) at a concentration of 4% (*w*/*v*). Both crosslinkers were dissolved in appropriate concentrations in 70% ethanol to obtain a final ratio of 1:0.8 of thiol- vs. acrylamide-groups in the resulting mixtures [17]. HA-SH and crosslinker solutions were gently but thoroughly mixed in a ratio of 7:3 to obtain a homogeneous gelation solution. Thus, hydrogels contained a final 2.8% HA-SH concentration immediately after crosslinking. Gelation by thiol-Michael addition was carried out in small cylindrical Teflon molds (r = 3 mm, h = 3 mm), sealed by glass slides for 24 h at room temperature. Prior to further experiments, hydrogels were swollen in PBS for 48 h to reach their swelling equilibrium [17].

To measure the number of free thiols in the hydrogels an adapted Ellmann’s assay was used. To calculate the percentage of reacted thiols and therefore assess the efficiency of crosslinking, a gelation mixture without crosslinker was used as a reference containing 100% free thiols [17].

#### 4.2.3. Mechanical Measurements

For the mechanical characterization of swollen hydrogels, a uniaxial compression test between parallel plates in a NanoBionix Universal Testing System (MTS Systems, Eden Prairie, MN, USA) was performed. Therefore, stresses resulting from an applied strain of 0 to 10% were measured. Young’s moduli (*E*) were subsequently obtained from this data by a linear fit in the linear-viscoelastic-region between 0 and 5% (Table 3) [17].

### 4.3. Calcultation of Negative Network Charge

Hydrogels consist of water, a polymer network and respective counter ions. We therefore simplify and define the negative network charge based on the contribution of HA and crosslinker only [17].

$$n\left(HA\;disaccharide\right)\;=\;\frac{m\;\left(HA-SH\;per\;hydrogel\right)}{M\;\left(HA\;disaccharide\right)}$$

$$n\left(crosslinker\right)\;=\;\frac{n\;\left(HA\;disaccharide\right)}{2}\;\times \;0.8$$

For uncharged crosslinker:

n(negative charge per hydrogel) = n (HA disaccharide) × (1−degree of thiolation)

For charged crosslinker:

n(negative charge per hydrogel) = [n (HA disaccharide) × (1−degree of thiolation)]− [n (crosslinker) × (degree of thiolation)]

### 4.4. Enzymatic Degradation

HA hydrogels were degraded in 1 mL of 0.005 µmol/mL hyaluronan degrading enzymes, hyaluronidase IV, and lyase. HA hydrogels in 1 mL PBS and 1 mL 0.005 µmol/mL trypsin were used as controls. For degradation, HA hydrogels were incubated in the corresponding solutions at room temperature and 100 rpm while enzyme and buffer exchange was carried out every 48 h. The weight loss of the hydrogels was continuously monitored over four weeks. From the obtained weight measurements, the half-lives of HA hydrogels could be calculated by fitting the data to an exponential decay phase 1 “GraphPad Prism7” (for Mac, version 7.0c, GraphPad Software Inc., La Jolla, CA, USA).

### 4.5. Protein Adsorption

The amount of adsorbed protein on the HA hydrogels was determined after incubation of the hydrogels with 50 µg/mL solutions of fibronectin, collagen, and aggrecan in PBS for 24 h at 37 °C. Subsequently, the hydrogels were thoroughly washed, before the adsorbed protein was dissolved with 1 mL of a 1% (*w*/*v*) SDS solution in PBS for 24 h at room temperature and 350 rpm. The protein concentration in the supernatant was then determined by BCA assay, using hydrogels incubated in pure PBS in the first incubation step as a blank.

### 4.6. Cell Cultivation on HA Hydrogels

Custom-made glass well plates (see Supplementary Materials, Figure S4) were pre-passivated with PLL(20 kDa)-g[3.5]-PEG(2 kDa). For this purpose, activation was carried out in an oxygen plasma for 10 min at 150 W before 0.25 mg/mL PLL(20 kDa)-g[3.5]-PEG(2 kDa) in 4-(2-hydroxyethyl)-1-piperazineethanesulfonic acid (HEPES) buffer, (10 mM, pH = 7.4) was added to each well. Passivation occurs within 45 min at room temperature. Subsequently, the glass wells were rinsed with ddH_2_O, the hydrogels were added into the wells, and the well plate with the hydrogels was sterilized for 30 min under UV light.

All three cell types (NHDF, HDLEC, and MCF7) were suspended in their respective media to allow seeding of 75,000 cells/hydrogel in a volume of 300 µL each. The pre-passivated glass well plate with different HA hydrogels and added cell suspensions was then incubated for 24 h at 37 °C and 5% CO_2_ to allow cell attachment.

For inhibition experiments, cells were blocked either with HCAM CD44 antibody or short HA in a concentration of 100 μg/mL for 30 min in suspension at 37 °C and 5% CO_2_. Subsequently, the cells were seeded in the same density as unblocked cells and treated equally for incubation.

### 4.7. Phase Contrast and Fluorescence Microscopy on HA Hydrogels

#### 4.7.1. Live/Dead Staining

After cells could adhere to the HA hydrogels for 24 h, non-specifically attached cells were washed from the hydrogel surface with PBS. For the attached cells, cyto-dye (excitation maximum: 488 nm; emission maximum: 518 nm) was used to stain live cells, whereas propidium iodide (excitation maximum: 488 nm; emission maximum: 615 nm) was used to stain dead cells [26]. For staining, the hydrogels with cells were incubated with 200 µL each of a solution with 1 µM cyto-dye and 1 µM propidium iodide in the kit’s staining solution for 15 min at 37 °C.

#### 4.7.2. Microscopy Imaging

Hydrogels were imaged with the inverted microscope “Observer Z1” in combination with the “Zen2pro” software from Zeiss. After adjustment of the light path according to the Koehler method [27], phase contrast images of the cells on the hydrogel surfaces were taken with a 20× objective (EC-Plan Neofluar, num0.5 NA, PH2) from Zeiss. Additionally, fluorescent images were taken with the green fluorescent protein (live cells) and rhodamine (dead cells) filter set. To determine the number of live cells on different HA hydrogels, composite images of phase contrast and green fluorescent protein channel images were created in "Fiji" (ImageJ 1.51 h) and live cells per image were counted manually and converted to cells/mm^2^, taking image size into account.

## Figures and Tables

**Table 1:** Bicinchoninic acid assay (BCA assay) of proteins on HA hydrogels (with degrees of thiolation in % = 18, 25, and 33 and uncharged and charged crosslinkers) shows no adsorption of collagen, fibronectin, or aggrecan after the subtraction of the phosphate-buffered saline (PBS) blank.

Degree of Thiolation (%)	Adsorbed Collagen (ng/mL)	Adsorbed Fibronectin (ng/mL)	Adsorbed Aggrecan (ng/mL)
18 ± 1	0.001 ± 0.019	−0.020 ± 0.011	−0.011 ± 0.019
25 ± 4	−0.015 ± 0.004	−0.029 ± 0.006	−0.024 ± 0.007
33 ± 2	−0.020 ± 0.004	−0.023 ± 0.012	−0.031 ± 0.004

**Table 2:** Reaction conditions and corresponding degree of thiolation for 74 kDa HA (Lifecore) used for the HA hydrogels presented in this paper.

Ratio of HA:DTP:EDCl	Batch of HA (Lot Lifecore)	Reaction Time	Degree of Thiolation
1:1:1	025828	10 min	18 ± 1%
1:1:1	025828	15 min	25 ± 1%
1:1:1	025828	20 min	33 ± 2%

**Table 3:** Mechanical properties of HA hydrogels with 74 kDa HA at three different degrees of thiolation with the uncharged or charged crosslinker show trends according to the two different chemical interactions (1) and (2). Values represent mean ± SD of three individual measurements [17].

Degree of Thiolation (%)	Crosslinker	Young’s Modulus (kPa)
18 ± 1	uncharged	0.36 ± 0.02
18 ± 1	charged	2.57 ± 0.26
25 ± 4	uncharged	2.68 ± 0.13
25 ± 4	charged	3.65 ± 0.71
33 ± 2	uncharged	3.20 ± 0.12
33 ± 2	charged	6.57 ± 0.71

## Supplementary Materials

The following are available online, Figure S1: Determination of half-life, Table S1: Determination of half-life, Figure S2: Unspecific degradation in PBS and trypsin, Figure S3: Results of BCA assay, Figure S4: Custom-made glass well plates for cell experiments, Figure S5: Cell attachment–passivation control, Figure S6: Cell attachment–images of life-dead-staining, Figure S7: Cell attachment–cell numbers with respect to negative network charge.
