# Supplementary material for: Charge-Controlled Synthetic Hyaluronan-Based Cell Matrices
Source: Molecules. 2018 Mar 27;23(4):769. doi: 10.3390/molecules23040769 (PMC6017843; doi:10.3390/molecules23040769)
Supplement: Supplementary file 1 [file molecules-23-00769-s001.pdf]

6. Supplementary Materials

6.1. Determination of half-life

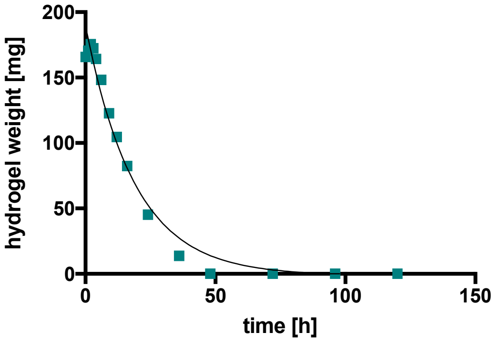

Figure S1. Weight loss of the hydrogels over time shows an exponential decay. From the exponential fit, half-life can be calculated with „GraphPad Prism7" (for Mac, version 7.0c, GraphPad Software Inc., USA). In this graph, a hydrogel with 25 % thiolated HA and the charged crosslinker is shown.

Table S1. Fit values for the first order exponential decay of hydrogel weights shown in Figure S1, determined with „GraphPad Prism7" (for Mac, version 7.0c, GraphPad Software Inc., USA).

Fit parameter	Value and Standard deviation
Best-fit Y0	189.3 ± 4.0
Best-fit Plateau	- 1.25 ± 1.71
Best-fit K	0.05267 ± 0.00333
Best-fit Half-Life	13.16 ± 1.74
Best-fit Tau	18.98 ± 2.50
Best-fit Span	190.6 ± 4.2
Equation	Y = (189.3 + 1.25) x $e^{-0.053x}$ – 1.25
Degrees of Freedom	28
R square	0.9891
Absolute Sum of Squares	1543

6.2. Unspecific degradation in PBS and trypsin

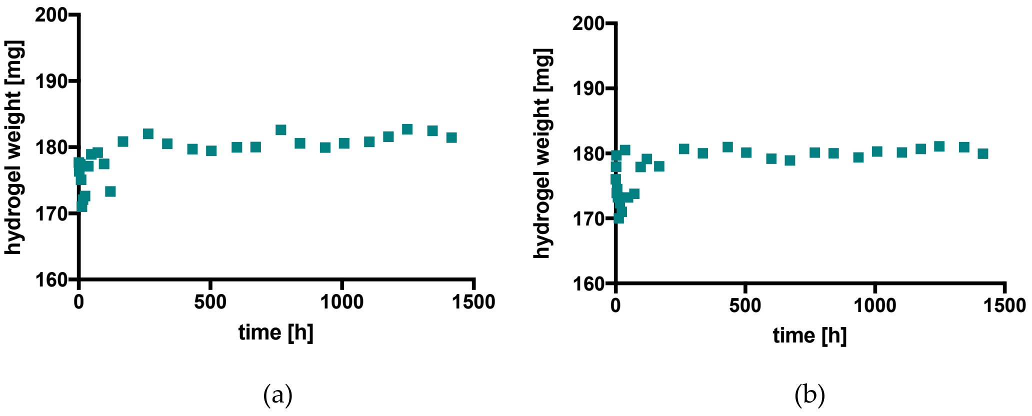

Figure S2. Hydrogel weight is constant in (a) PBS and (b) trypsin solution over the course of eight weeks. Thus, no unspecific degradation of hydrogels in solution can be observed.

6.3. Results of the BCA-assay

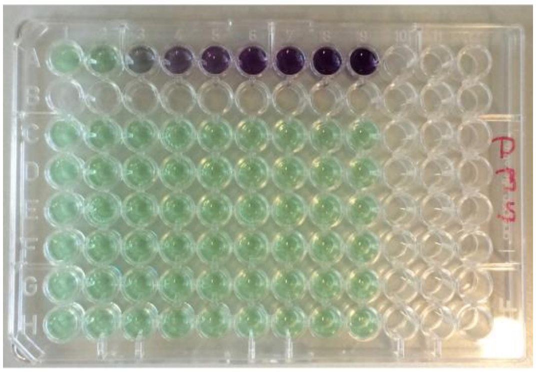

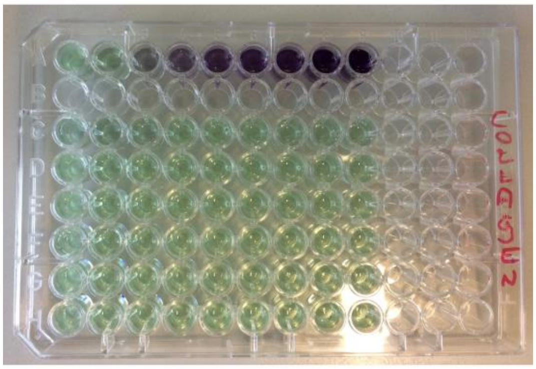

(a) (b)

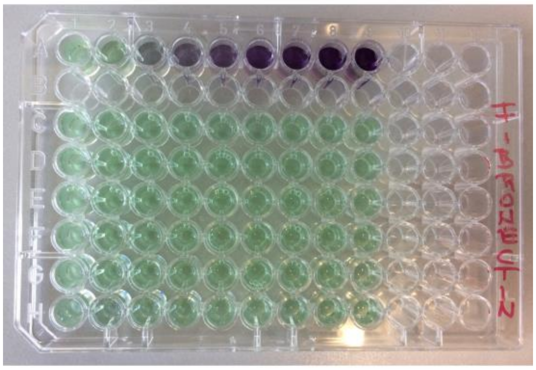

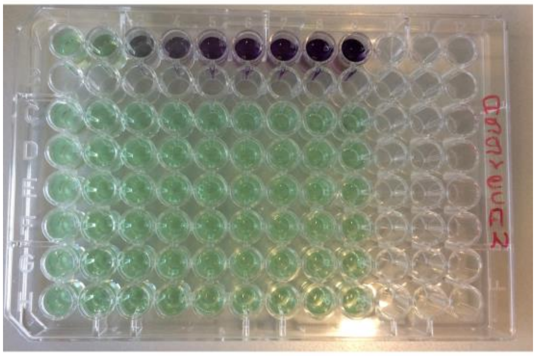

(c) (d)

Figure S3. Hydrogels incubated with (a) PBS, (b) collagen, (c) fibronectin and (d) aggrecan show no adsorbed protein in any of the tested conditions. In each image lane C-E are hydrogels with the uncharged crosslinker and 18% thiolated HA (C), 25% thiolated HA (D) and 33% thiolated HA (E) respectively, whereas F-H are hydrogels with the charged crosslinker and 18% thiolated HA (C), 25% thiolated HA (D) and 33% thiolated HA (E).

6.4. Custom made glass-well plates for cell experiments

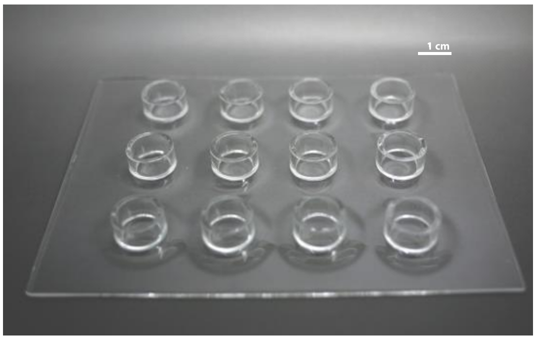

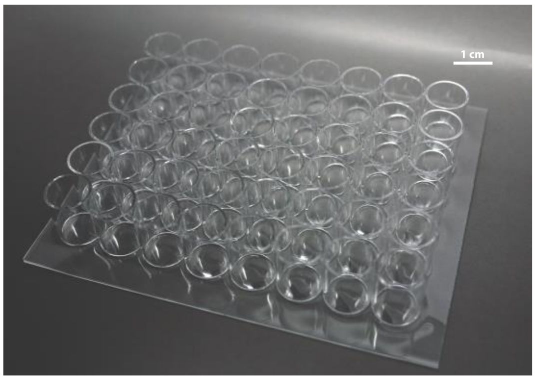

(a) (b)

Figure S4: Custom made glass-well plates used for cell seeding on HA hydrogels. They can be passivated with PLL-g-PEG to prevent unspecific cell adhesion to the glass background. Scale bars represent a length of 1 cm.

6.5. Cell attachment – passivation control

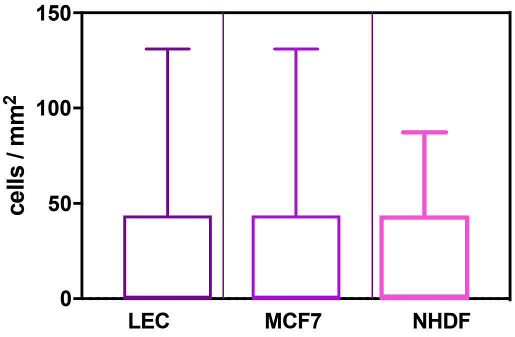

Figure S5. Small numbers of all three cell types HDLEC, MCF7 and NHDF are able to attach to PEG passivated coverslips. All values represent mean and standard deviation of 30 individual images of three PEG passivated surfaces and 10 images per surface. Cell numbers are counted manually after manual adjustment of each individual image.

6.6. Cell attachment – images of live-dead staining

With a live-dead double staining kit, viable and dead cells are simultaneously stained. The kit utilizes the non-fluorescent acetoxymethyl ester of calcein (calcein-AM), which is highly lipophilic and thus cell membrane permeable, to stain viable cells. The observed strong green fluorescence (λ_{ex} = 490 nm, λ_{em} = 515 nm) of these cells is induced by calcein, generated by esterase in viable cells. Therefore, calcein-AM only stains viable cells.
For staining dead cells, the nuclei staining dye propidium iodide (PI) is used as it cannot penetrate the cell membrane of viable cells, but passes through disordered areas of dead cell membranes. Red fluorescence (λ_{ex} = 535 nm, λ_{em} = 617 nm) is generated by intercalation of PI with the DNA double helix in the cell nucleus of dead cells.

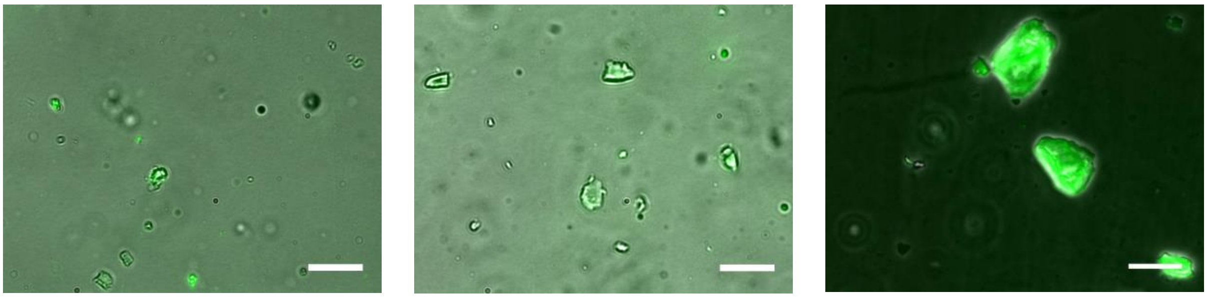

(a) (b) (c)

Figure S6. (**a**) HDLEC, (**b**) MCF7 and (**c**) NHDF are alive on HA hydrogels, with a degree of thiolation of 33 % and the charged crosslinker used as an example in the pictures. Phase contrast images are shown in black and white, overlayed with green fluorescence images of the live/dead cell staining. Scale bars represent 25 µm in all three images.

6.7. Cell attachment – cell numbers in respect to negative network charge

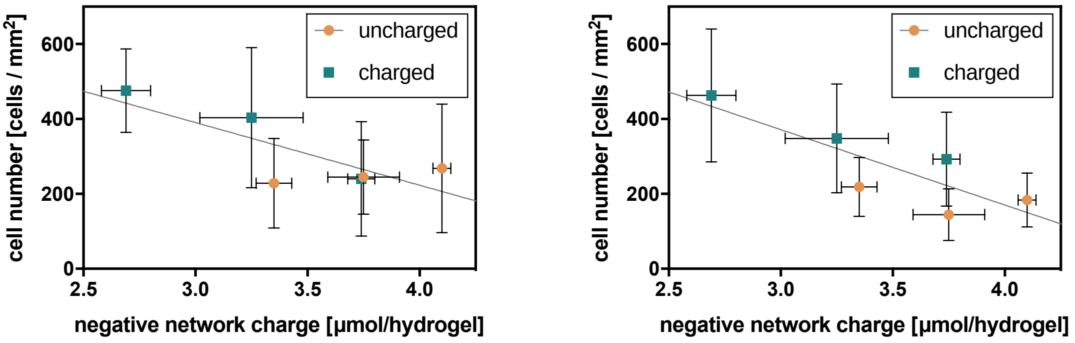

(a) (b)

Figure S7. Number of MCF7 (**a**) and NHDF (**b**), attaching on HA hydrogels (with degrees of thiolation in % = 18, 25 and 33 and uncharged and charged crosslinker) is dependent on the negative network charge of the ECM mimetic. Both MCF7 (**a**) and NHDF (**b**) show a linear correlation of negative network charge and number of cells attaching to the different HA hydrogels.
